# Case Illustrating the Effects of a Vegetable Poison

**Published:** 1814-11

**Authors:** J. Adam

**Affiliations:** Forfar


### *For the Medical and Physical Journal.*

### *Case illustrating the Effects of a Vegetable Poison;* by Mr. J. Adam, jun. of Forfar.

ON Saturday, the 9th of October, 1813, at six, p. m. a tradesman of this place came to me in great distress, and begged that I would immediately visit his daughter, a girl of eight years, whom he declared to be poisoned by eating mushrooms. She had eaten one fungus on the preceding evening, between seven and eight; and on Saturday, at three, p. m. six more of the same species, of a middling size; the membrane which covers the upper surface had been removed from all of them: they were dressed with salt an hour before being used, and afterwards roasted at a common fire. Having taken no dinner previously, she devoured them greedily. She continued apparently quite well, running about as usual, until a few minutes before five, when, after having taken tea, she complained slightly of a soreness affecting the belly; and, hastily eating an apple which her mother gave her, on moving across the room she cried out that she felt herself giddy, and instantly fell on the floor, deprived of sense and motion. When carried to bed she uttered a wild cry, and the attendants remarked a peculiar fierceness of the eyes.

Some ipecacuanha wine had been administered, and she also drank about two English pints of warm water, but without any vomiting. I dissolved 25 grains of sulphate of zinc in a small quantity of water, and ordered it to be given without delay, which, as I found on visiting her a little afterwards, had been swallowed without effect. When I first saw her (about a quarter past six) she was stretched out in bed in a comatose state, with the countenance somewhat swollen, and of a ghastly leaden aspect; pulse greatly intermittent and tremulous, and so weak as scarcely to be felt, with the eye-balls pulled up towards the angles of the orbits, and fixed immoveably in their sockets, affected now and then with startings of the limbs, and severe convulsive motions of the head and upper part of the trunk. She appeared altogether unconscious of what was passing around her, and nothing was expected by the by-standers but speedy dissolution. Indeed all the symptoms strongly marked the danger of the case, and shewed in the most unequivocal manner the effect of some deleterious agent exerting a powerful influence on the system in general. An unusual degree of coldness prevailed over the whole body, and the heart laboured greatly in its action. On seeing the condition she was in, I requested a little cold water, which was dashed dashed from the distance of a few feet on the naked breast. This application produced the stimulant effect I had looked for; and making a sudden start, which raised the head from the pillow, the patient opened her eyelids, and for a few seconds stared wildly around. Recollecting the observations of Richerand on the *ultimum moriens* of the human body, and his recommendation of stimuli to the gut in cases where the powers of life are low; I then ordered a clyster, consisting of 25 grains of camphor made up into an emulsion, with a little gum arabic mucilage and water; and, as no vomiting had as yet ensued from the first quantity of sulphate of zinc, half a drachm more was at the same time given, dissolved in water as before, and it was also attempted to assist the action of the emetic, by tickling the fauces, and compression with a bandage over the epigastric region; heated flannel cloths were applied in quick succession to the surface; a free evacuation followed from the injection, but had no appearance of fungi intermixed. From the power of swallowing being already much impaired, it was with considerable difficulty that the second emetic solution was got over, and only by repeated efforts, after rousing our patient by the application of water of ammonia to the nostrils and mouth; and, although the greater part appeared to have been received into the stomach, she only once shewed an inclination to vomit, and a little mucus merely mixed with saliva was ejected.

The agitation produced by this, however, seemed to have had a good effect in rousing her, but she quickly relapsed into her former lethargic condition, and the symptoms always increased; the convulsions becoming more violent, longer in their duration, and recurring at shorter intervals, and the coldness and rigidity in the extreme. All attempts to discharge the noxious matter were now abandoned as unavailing, and our whole care was directed to counteracting the pernicious effects of the poison, whose operation had but too clearly manifested itself.

The practice which had in part been begun, was most strenuously persisted in, and however desperate the case appeared, I determined on persevering, and to wait the result. Equal parts of aq. ammoniæ and water were mixed together, and of this she had two or three tea spoonfuls every eight or ten minutes, and occasionally in the intervals, or alternated with it, such quantities of rum and water as she could be got to swallow. The aq. ammoniæ simply was frequently rubbed on the face and about the eyes and mouth, which affected her considerably, and generally produced a momentary flushing of the countenance. Its employment in this mode mode we always premised, as facilitating the exhibition of the other remedies; and, taking advantage of the mouth being open from the cough or sneezing which was excited, we poured the liquids into the stomach, which, from the rigidity of the jaw, and palsied condition of the muscles of the pharynx and gullet, could not otherwise have been well accomplished; and, even with the assistance of this, we seldom could get the full dose introduced, some being generally lost from the involuntary motions of the head during the time of administering it.

The warmth of the body was attempted to be restored by the continued application of heated flannels, as at first employed; and by means of the same, the whole surface was frequently rubbed with common salt, and also with aq. ammoniæ. I dashed cold water in small quantity four different times on the breast, and each application was succeeded by a violent movement of the upper part of the body, and a wild kind of scream, and the pulse became for a little more regular and fuller.

About an hour after the first injection, an ounce of common turpentine was made into a clyster, and one-half thrown up the rectum, which produced a copious stool, rather fetid, but having no appearance of fungi. She was often violently shaken, but without any sensible effect. Nitre was largely decomposed around the patient in red-hot shovels, (the only instrument that offered itself,) and at times, instead of the hartshorn and water, the alcohol ammoniatum, diluted and mixed with ether, and wine and water, with a portion of powdered capsicum, were substituted; all which, as before, we still continued to administer, at an interval of about ten minutes. The pulse now altered much, and from being intermittent became regular, but weak and quick, being upwards of 130.

During the employment of all these means, which might have occupied the space of three hours or a little more, excepting the change in the state of the circulation, we perceived no favourable symptom that might lead us to look for a happy termination of the case; but to have our patient alive after that lapse of time was more than appearances at first warranted us to expect, and we were encouraged to continue the practice, although no positive mitigation of the symptoms had as yet taken place. She was then taken out of bed, and put up to the neck in a large stand full of warm water, (the vessel being inclined as much as could be done to the horizontal position,) and kept immersed in it for ten minutes. After removal from the bath, the pulse became fuller and less quick, being reduced to 80 strokes in the minute. A mustard poultice was then applied to the region of the stomach and one to the sole of each foot. Soon after a pretty general perspiration broke out, and the heat of the body was also sensibly increased to the feel, though yet below the standard of health, the extremities were even more convulsed, and the muscles of the calf became permanently contracted, and felt under the hand rigid and hard; but the upper part of the body was much less affected than at first. Some tincture of soap and opium, with a large proportion of this last, was rubbed in over the limbs, and the second half of the turpentine injection was administered, but flatus merely accompanied its evacuation. The pulse got quicker again, and indeed seldom continued for half an hour steady, but the breathing never was laborious, and did not appear to deviate from the natural state, with the exception of a strange sound once or twice emitted. A fourth clyster which was thrown up brought off some excrement nowise unusual in its appearance. After remaining for two hours, the mustard cataplasms were removed, and it was found that they had produced a beginning derivation to the surface, the parts being highly reddened, but without any vesication. Some colour now appeared in the face, and she seemed to be much better. The starting of the limbs recurred less frequently, and at twelve I considered her out of danger. The skin was moist, and the body having much more of the natural heat; the pulse still about 125 or 130. We then relaxed in the application of the stimulant remedies, and, on leaving her to the charge of an assistant for about twenty minutes, on my return I found she had continued the same, another clyster having been administered in my absence, without any dejection following. She was to all appearance as a person in a state of sound sleep, breathing easily, with the countenance a little flushed, and the convulsive motions affecting her at longer intervals. Seeing now no farther occasion for a continuance of that practice which we had all along pursued, I recommended that she should not be disturbed, and warm flannels only applied from time to time to the trunk and lower extremities.

By the account of a sensible attendant who sat by her, she continued in the same profound sleep, without any manifest change, unless that a considerable perspiration broke out, until twenty minutes past five next morning, when she first opened her eyelids, and turned the eye in the orbit, seeming to look around her without any consciousness of perception. She then fell back again, and dozed until a quarter before seven, when suddenly starting up she threw down the bed-clothes, calling out at the same time to take them off from from her, and seemed perfectly sensible. Being supported on the side of the bedstead, she vomited up more of the fungi than would have filled two moderate-sized teacups, not altered in appearance from the state in which they had been received into the stomach. She then asked for something to drink, and complained much of a soreness of the head and neck, so that she could not swallow. She afterwards vomited more, and retched a good deal, without any matter being discharged. When I saw her in the morning, the pulse beat 120 strokes in the minute, weak but regular. Some tea was offered her, which she would not take. Two drachms of the compound powder of jalap were ordered, a third to be taken every hour until the bowels should be freely moved, and infusion of tamarinds for drink. The two first doses of the powder were vomited a short time after being taken, and the tamarinds she could not be induced to use. In the course of the day she took some wine and water, and, as no evacuation of the bowels had been procured, a clyster of senna and Glauber's salts was administered, which produced a stool rather fetid, and having an evident intermixture of the fungi, with the laminæ entire. She ate nothing all the 10th, and the belly continued loose, each stool containing some portion of the mushroom in an undigested state, and she was affected every now and then in the course of the day with involuntary motions of the lower extremities. Thirst considerable, pulse in the afternoon 110. During the night she slept nearly as usual, but was frequently affected with startings. On the 11th she took some breakfast with considerable appetite; pulse 100, small. During the two succeeding days she continued pretty well, but on the 14th the pulse was irregular, and she had been passing still some remains of the mushroom; a laxative being given, which produced several evacuations, she continued free from complaint.

A brother of the above, a boy of eight years of age, had eaten one on the Friday, but after dinner; and, before five the same evening, he was seized with vomiting, and the fungus being rejected no bad consequence ensued. Another brother, of nine years, had eaten also one, and during the night was affected with soreness of the belly; but, notwithstanding he had taken some more on the ensuing day, he was not attacked with any bad symptoms, as in the case of his sister.

James Boath, of four years, had also eaten some on Friday quite raw, but the quantity was unknown. About seven in the evening of the same day, he was seized with pain of the belly, which continued during the whole of next day, until an emetic which he took on Sunday brought off the fungi with with the membrane covering the upper part, and apparently in the same state as when swallowed.

James Tuck, aged three years, ate some of the same species in the wood on Friday. He was taken ill the same evening, vomited and purged much, belly swollen and tense, and cold sweats broke out on different parts of the surface, and the body in general felt below the standard temperature of health, and was affected with great thirst. He went about, however, on Saturday, and ate some more, and was taken ill again in the evening with vomiting and purging; and, an emetic of ipecacuanha being given, a quantity of the fungi was discharged, but for several days after he loathed his food. His elder brother, Stephen, ate one; but an emetic having been administered, no bad consequences ensued.

In one only of the cases, that of Boath, did the poison produce a permanent effect. During all the winter the boy suffered from a diarrhœa, and frequent convulsive affections, particularly of the superior extremities and face; he became emaciated and pale, and lost all relish for food. In the course of the last summer he has regained his health, and is now quite stout.

The species of mushroom which had been used by all the children is that described by Lightfoot in his *Flora Scotica*, under the title of *Muscarius*, and now flourishes in our woods in great abundance. It is a beautiful fungus, and the tallest to be met with, of a red colour at top, with small specks of a whitish matter, the remains of the involucrum, which surrounds the whole before it shoots from under the turf. It is almost insipid; and, in this respect as well as in its general appearance, differs widely from another species, the *Integer*, which also grows in great plenty in our neighbourhood, and in the same situations as the muscarius. The colour of the integer is purple, or sometimes inclining to crimson, of a most pungent biting taste, so much so that the sensation remains on the tongue for many hours after its application. This species, which I should conceive from its sensible qualities to be the more deleterious, had not been taken in the smallest quantity; and the effects exemplified in the case of Isabel Thorne, (as above narrated,) are to be referred entirely to the other species, the muscarius. The practice employed in her case was such as the symptoms suggested; but, if hereafter a similar one should occur, I should certainly carry the use of stimulants to a greater extent, and have recourse at first to the warm bath, the benefit of which was particularly conspicuous. Perhaps stronger emetics might have been used, and a flexible tube would have forwarded greatly the exhibition of our internal means. In such cases where the powers of the system are low, and the introduction of medicines by the mouth difficult, might not the pneumatic art be of considerable avail, and the inhalation of stimulating gases affect materially the other means in use? At any rate, remedies possessing the same general virtues, when applied in combination, must be much more powerful than when singly or in succession; and it was with this view, that while all the other avenues (the senses perhaps excepted) by which the system could be affected, the stomach, the skin, and the gut, were taken advantage of, that I ordered the decomposition of the nitrate of potass, by which also the lungs might receive a greater portion of oxygen, and the heart be excited to more vigorous action by its proper stimulus. In some experiments which I made on dogs, with an extract prepared by expression of the fungus, and inspissation of the juice on a slow fire, no manifest noxious effect was induced; a slight purging in one only followed its exhibition.—The aq. ammoniæ used above is a diluted preparation, and sold in the shops under the old name of spirits of hartshorn; it is however pure, and not a carbonate.

*Forfar, N.B.* JOHN ADAM, jun.
*Sept.* 21, 1814. Surgeon.

